# First report of *Leishmania* (*Viannia*) *lindenbergi* causing tegumentary leishmaniasis in the Brazilian western Amazon region

**DOI:** 10.1051/parasite/2019030

**Published:** 2019-05-23

**Authors:** Lilian Motta Cantanhêde, Cristiane Batista Mattos, Camila de Souza Ronconi, Camila Patrício Braga Filgueira, Cipriano Ferreira da Silva Júnior, Claudino Limeira, Helen Paula de Jesus Silva, Gabriel Eduardo Melim Ferreira, Renato Porrozzi, Ricardo de Godoi Mattos Ferreira, Elisa Cupolillo

**Affiliations:** 1 Fundação Oswaldo Cruz, Unidade Rondônia Porto Velho Rondônia Brazil; 2 Fundação Oswaldo Cruz, Instituto Oswaldo Cruz, IOC Rio de Janeiro Brazil; 3 Secretaria de Saúde de Rondônia, SESAU Porto Velho Rondônia Brazil

**Keywords:** *Leishmania* (*Viannia*) *lindenbergi*, Tegumentary Leishmaniasis, Western Amazon

## Abstract

Tegumentary Leishmaniasis (TL) in the Brazilian Amazon region is associated with several *Leishmania* species. In this report, we describe two cases of TL related to *Leishmania lindenbergi* occurring in different locations of Rondônia state. After clinical diagnosis, lesion samples were collected for parasitological diagnoses via direct microscopic visualization, parasite isolation, and PCR. PCR reactions were positive in both clinical samples. Parasite isolation was possible for both patients, and isolates were submitted to species identification by isoenzyme electrophoresis and DNA sequencing. This report is the first to describe human infections caused by *L. lindenbergi* since the initial description and record of human infection by this species in 2002.

## Abstract (fr)

La leishmaniose tégumentaire (TL) en Amazonie brésilienne est associée à plusieurs espèces de *Leishmania*. Dans ce rapport, nous décrivons deux cas de TL liés à *Leishmania lindenbergi* survenus à différents endroits de l’état de Rondônia. Après le diagnostic clinique, des échantillons de lésion ont été recueillis à des fins de diagnostic parasitologique par visualisation au microscope direct, isolement du parasite et PCR. Les réactions de PCR étaient positives dans les deux échantillons cliniques. L’isolement des parasites a été possible pour les deux patients et les isolats ont été soumis à l’identification de l’espèce par électrophorèse des isoenzymes et séquençage de l’ADN. Ce rapport est le premier à décrire les infections humaines causées par *L. lindenbergi* depuis la description initiale et le signalement de cas d’infection humaine par cette espèce en 2002.

## Introduction

Various *Leishmania* species act as etiological agents in Tegumentary Leishmaniasis (TL). More than 20 species of *Leishmania* are known to be infectious to humans. Some of these species are widespread around the world with major concentrations in tropical and subtropical regions. In Brazil, seven *Leishmania* species have been identified as human pathogens causing TL: one species of the subgenus *Leishmania* (*Leishmania*): *L amazonensis* and six species of the subgenus *Leishmania* (*Viannia*): *L. braziliensis*, *L. guyanensis*, *L. naiffi*, *L. lainsoni*, *L. shawi*, and *L. lindenbergi*. All of these species are endemic to the Brazilian Amazon region [9, 13, 14].

The rarest species registered in Brazil is *L. lindenbergi*, which was reported to cause human infections in soldiers performing activities in secondary forests and in a woman from the same area in Belém, state of Pará, Brazil [13]. This single report refers to human infections, but little is known concerning the parasite’s biology, including hosts, reservoirs, and vectors. The most likely associated vector is *Nyssomyia antunesi* due to the abundance of this vector in the area where the soldiers were infected by *L. lindenbergi*. The description of the clinical features, as well as the course of the infections caused by *L. lindenbergi*, are also limited, although all infections that were reported presented with localized cutaneous manifestations [13].

## Materials and methods

**Figure 1:**
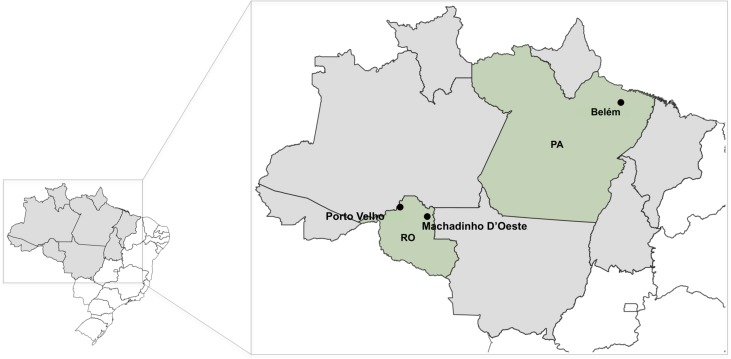
Map of Brazil showing the localities where *Leishmania* (*Viannia*) *lindenbergi* was already detected. The Amazon region is presented in grey, and in green are the two States (PA = Pará; RO = Rondônia) showing the municipalities that presented cases of human cutaneous leishmaniasis caused by *L. lindenbergi*.

During the period from 2013 to 2017, more than 500 patients with suspected Cutaneous Leishmaniasis were seen at the Rondônia Reference Hospital for Tropical Medicine (CEMETRON). In August 2014, a 32-year-old male patient (Patient 1) presenting cutaneous lesions for approximately 1 year and 8 months that were located on the left arm, was seen at CEMETRON. The patient reported that the infection was acquired in a rural area of the municipality of Machadinho D’Oeste, in the state of Rondônia, where he lives and works in agriculture. This municipality is approximately 400 km from Rondônia’s capital, Porto Velho (Fig. 1).

**Figure 2:**
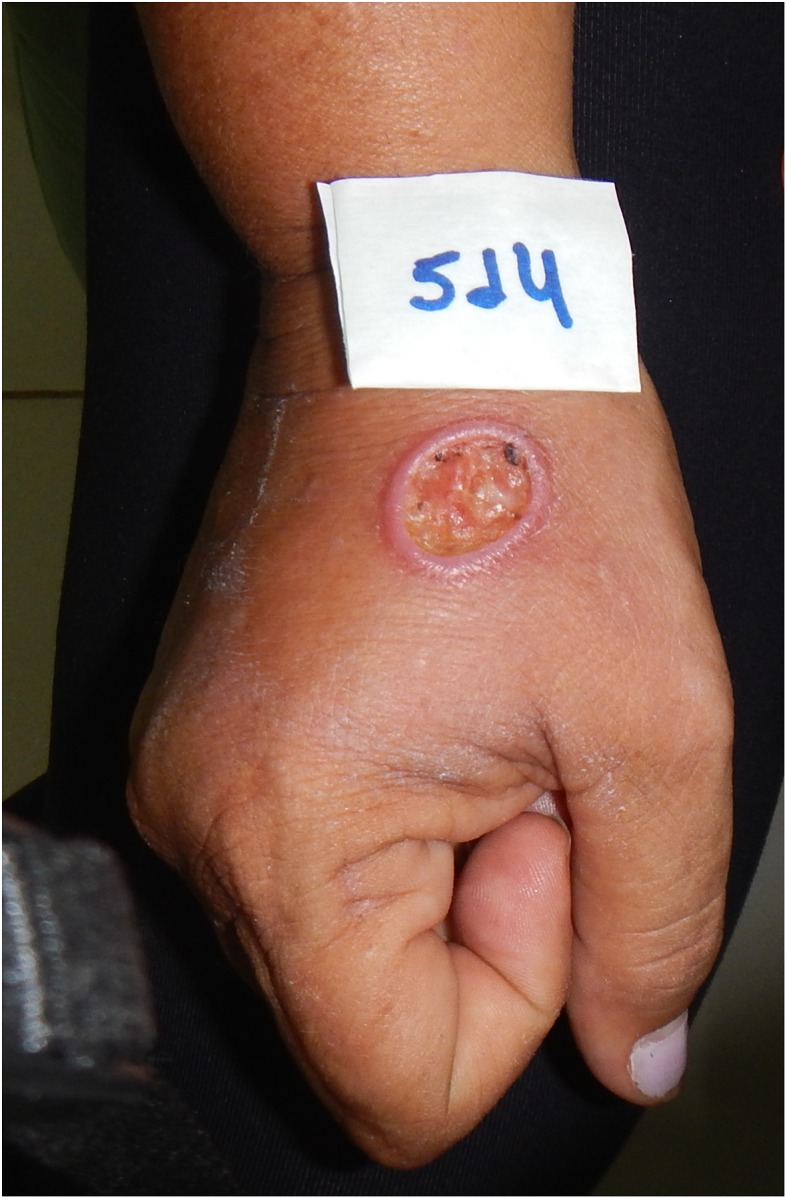
Single cutaneous Leishmaniasis lesion associated with *Leishmania lindenbergi* observed in a patient (Patient 2) from the State of Rondônia, Brazil. Clinical diagnosis was confirmed by direct and indirect parasitological tests (see Table 1 for details), and *Leishmania* species identification was achieved by Multilocus Enzyme Electrophoresis (MLEE).

In December 2015, another patient, a 50-year-old woman (Patient 2), with a cutaneous lesion on the left hand (Fig. 2) was seen at CEMETRON. She reported that the infection may be related to labor activities and was acquired on “Estrada do Índio” (Federal Road 319), a federal highway that connects the cities of Porto Velho and Humaitá, in the Brazilian states of Rondônia and Amazonas, respectively (Fig. 1). This highway has an extension of approximately 900 km, is surrounded by Amazon forest, and is an area with frequent reports of TL.

Clinical data and biological samples were collected within the scope of the research project conducted at CEMETRON, and this study was approved by the Ethics Committee under the protocol CAAE 0020.0.046.000-11. Patients were informed about the research project, agreed voluntarily to participate, and signed consent forms. The two patients did not report any chronic disease and the investigation for HIV coinfection was negative. Parasite isolation was performed by inoculating the lesion border aspirate in biphasic culture medium (NNN + Schneider supplemented). Samples for molecular detection and identification of the parasite were collected using sterile cervical brushes placed in direct contact with the edge of the lesion. PCR was performed targeting *k*DNA [6] and *hsp*70 [5] for *Leishmania* detection.

## Results and discussion

Positive results were obtained in all parasitological tests for samples from Patient 2, while for samples from Patient 1, only PCR targeting *k*DNA and parasite isolation in culture medium were positive (Table 1). *Leishmania* parasites were observed for both isolates in less than 30 days and both cultures reached the amount of parasites needed for cryopreservation approximately 20 days after the first parasite visualization. Both isolates were negative for the presence of the viral endosymbiont *Leishmania RNA Virus* 1, as determined following protocols described elsewhere [4].

**Table 1:** Description of parasitological tests performed for samples collected in this study.

Sample	Microscopy	PCR *k*DNA	PCR *hsp*70	Parasite isolation	International code
Patient 1	NEG	POS	NEG	POS	MHOM/BR/2014/RO285
Patient 2	POS	POS	POS	POS	MHOM/BR/2015/RO514

NEG, negative; POS, positive.

Patient 1 did not return to perform the treatment at CEMETRON hospital and the second patient obtained clinical cure 90 days after the treatment recommended by the Ministry of Health from Brazil (Glucantime^®^ 15 mg/day for 20 days).

**Figure 3:**
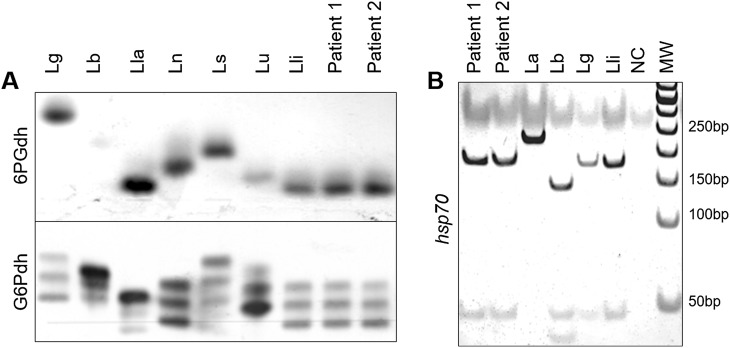
Multilocus Enzyme Electrophoresis (MLEE) and *hsp70* PCR-RFLP for *Leishmania* species identification. (A) Agarose gels stained for activity of 6-phosphogluconate dehydrogenase (6PGD) and glucose-6-phosphate dehydrogenase (G6PD) showing the patterns for *Leishmania* parasites isolated from Patients 1 and 2. (B) Polyacrylamide gel showing *hsp*70 products digested with *HaeI*II for *Leishmania* parasites isolated from Patients 1 and 2. In both assays, the profiles obtained for the two samples were compared to reference strains from different *Leishmania* species. For MLEE, the pattern was compatible with *Leishmania* (*Viannia*) *lindenbergi*. *hsp70* PCR-RFLP was not useful for species identification of parasites from the two patients studied, as this marker cannot distinguish between *L*. (*V*.) *lindenbergi* and *L*. (*V*.) *guyanensis*. Lg = *L*. *guyanensis*, Lb = *L*. *braziliensis*, Lla = *L*. *lainsoni*, Ln = *L*. *naiffi*, Ls = *L*. *shawi*, Lu = *L*. *utingensis*, Lli = *L*. *lindenbergi*, and La = *L*. *amazonensis*; NC = Negative Control; MW = Molecular Weight

Isolated *Leishmania* were deposited at the *Leishmania* Collection of the Oswaldo Cruz Institute (CLIOC) and processed for identification at the species level, employing multilocus isoenzyme electrophoresis (MLEE). Samples were identified as *Leishmania* (*Viannia*) *lindenbergi* (Fig. 3A) and deposited in CLIOC as IOC/L3645 (MHOM/BR/2014/RO285) and IOC/L3746 (MHOM/BR/2015/RO514).

**Figure 4:**
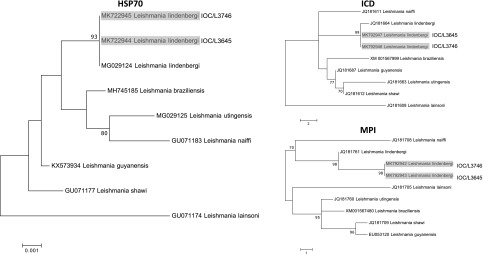
Neighbor-joining trees based on the analysis of partial sequences of *hsp70*, icd and mpi for *L*. (*Viannia*) species, indicating the identity of IOC/3645 and IOC/L3746 with *L*. *lindenbergi*. Bootstrap test (1000 replicates) was performed and values above 70% are shown. The trees are drawn to scale. The evolutionary distances were computed using the number of differences method [8]. GenBank accession numbers for each sequence are presented before the name of the species corresponding to each branch.

The positive *hsp*70 sample (Patient 2) was submitted to RFLP for *Leishmania* species identification [5]. The profile obtained was compatible with species of the subgenus *Viannia,* but species identification could not be accomplished. PCR-RFLP of the *hsp*70 gene was performed employing DNA extracted from *Leishmania* cultures, the same as used for the isoenzyme assay, and the profiles obtained by *HaeIII* digestions were compatible with *L. lindenbergi* and *L. guyanensis*, as these species presented the same pattern for this marker using this approach (Fig. 3B). A larger portion of the *hsp70* gene was amplified and sequenced, following already described protocols [12]. Both sequences were deposited at GenBank under the accession numbers MK792944 (IOC/L3645) and MK792945 (IOC/L3746). The global alignment using BLAST indicated 98.84% and 99.80% of identity with the sequence MG029124, a *Leishmania lindenbergi* sequence publicly available at GenBank, for IOC/L3645 and IOC/L3746, respectively. Another two genes were partially sequenced, isocitrate dehydrogenase (ICD) and mannose phosphate isomerase (MPI) for the two samples, as both regions are useful to distinguish *L. lindenbergi* from the others *L*. (*Viannia*) species [1], and the identification of IOC/L3645 and IOC/L3746 was confirmed as *L. lindenbergi* (Fig. 4). For ICD, IOC/L3645 and IOC/L3746 showed 99.77% and 98.77% identity with *L. Lindenbergi* (GenBank accession number JQ181664), respectively. For MPI, the highest identity observed was also with *L. lindenbergi* (GenBank accession number JQ181761), 94.36% and 98.81% for IOC/L3645 and IOC/L3746, respectively. Cluster analyses were performed to show the similarity of both *L. lindenbergi* strains, from Rondônia State, with *Leishmania* (*Viannia*) species circulating in the Amazon region (Fig. 4).

To date, *L. lindenbergi* has only been observed in human infections within the Brazilian state of Pará (Fig. 1), as presented in a study describing this *Leishmania* species. Therefore, there is little information describing the biology of this parasite or its transmission cycle [13]. In the description of this species, a similarity was mentioned to others of the same subgenus, such as *L. naiffi*. The molecular protocols used in the present study can distinguish *L. lindenbergi* from *L. naiffi*, but the final identification as *L. lindenbergi* was possible through the analysis of isoenzymes and DNA sequences of *hsp70* and housekeeping genes, since PCR-RFLP of *hsp70* does not allow discrimination between *L. lindenbergi* and *L. guyanensis*, as already mentioned. The difficulty of properly identifying *Leishmania* species using the PCR-RFLP *hsp70* was recently described, highlighting a limitation of this approach in some circumstances [7]. As presented by Silveira et al. [13], it is possible that *L. lindenbergi* is associated with more cases of TL if we consider that some methodologies currently employed for *Leishmania* species identification may not distinguish all species.

Out of seven *Leishmania* species associated with human TL infections in the North Region of Brazil, only *L. lindenbergi* had yet to be recorded in the state of Rondônia [3, 4]. In this state, approximately one thousand new cases of TL are registered per year, and the municipalities of Porto Velho and Machadinho d’Oeste comprise 16% and 4% of these cases, respectively [2].

The most likely vector of *L. lindenbergi, N. antunesi,* is abundant in the rural areas of Porto Velho, representing 25% of collected sandflies [11]. Natural infection by *Leishmania* spp. were already reported for this species in a study examining phlebotomines collected inside caves from different areas of Rondônia [10].

The diversity of sand fly vectors and *Leishmania* species currently described in the Amazon Region hinders efforts to characterize TL. The impossibility of distinguishing between *L. lindenbergi* and *L. guyanensis* when using *hsp*70 PCR-RFLP, currently one of the most widespread molecular markers for *Leishmania* identification, must be taken into account. This issue affects the reports concerning species distribution in endemic areas, which is an important aspect for understanding the epidemiology of TL and is even more relevant in areas with various etiological agents.

Few studies have addressed the taxonomic status of *L. lindenbergi*, and a genetic relationship of this species with *L. naiffi*, a genetically polymorphic parasite, has already been demonstrated [1]. Performing further studies to investigate the origin and genetic relationships of poorly studied species, such as *L. lindenbergi*, is crucial. For now, we can state that parasites with the genetic profile of *L. lindenbergi* could be widespread in distinct regions of the Brazilian Amazon, but further investigations are needed to confirm the distribution and frequency of this species.
